# Editorial: Disparities in mental health

**DOI:** 10.3389/fpsyt.2024.1379324

**Published:** 2024-02-13

**Authors:** Mohsen Merati, Hamidreza Komaki, Farnam Mohebi, Hannaneh Kabir, Lauren M. Haack

**Affiliations:** ^1^ Division of Gastroenterology, Department of Medicine, University of California San Francisco (UCSF), San Francisco, CA, United States; ^2^ Khoury College of Computer Sciences, Northeastern University, San Francisco, CA, United States; ^3^ Hass School, University of California Berkeley, San Francisco, CA, United States; ^4^ Cellular and Molecular Biomechanics Lab, Department of Bioengineering, University of California at Berkeley, San Francisco, CA, United States; ^5^ Department of Psychiatry and Behavioral Sciences, University of California, San Francisco (UCSF), San Francisco, CA, United States

**Keywords:** mental health disparities, socioeconomic status, LGBTQ+ mental health, health-related quality of life (HRQoL), geriatric psychiatry, depression and anxiety, psychiatric rehabilitation, suicide prevention

Health inequities not only pose an economic burden but also challenge social justice principles. While much of our understanding of health disparities focuses on physical illnesses, mental health is particularly susceptible to discrepancies. Addressing mental health issues requires trust, cultural sensitivity, acceptance, and support from healthcare providers. Individuals facing mental health challenges encounter disparities akin to physical illnesses and additional obstacles like societal stigma and language barriers. Identifying specific areas of mental health inequity, determining affected demographic groups, and assessing policy effectiveness are crucial steps to reduce disparities and promote well-being.

Research Topic of Mental Health Disparities in the Journal of Frontiers in Psychiatry explores various aspects within diverse social groups, aiming to elucidate current discrepancies, identify determinants, and propose mitigation strategies. Disparities manifest in various forms, including race, gender, sexual identity, LGBTQ+ populations, age, disability, socioeconomic status, and geographic location.

This Research Topic aims to inspire, inform, and provide direction and guidance to researchers in this field (**Table 1**). Each paper specifically addresses a practical point for researchers to consider as they seek to expand their knowledge in the realm of mental health disparities.

**Table 1:** Articles in the Research Topic of disparities in mental health.

Authors	Title	Country of Origin	Aim/Purpose	Study	Number of Participants		Summary of Result	Interpretation
Amirova et al.	Perceived discrimination in middle-aged and older adults: Comparison between England and the United States	UK	Examination of differences in perceived discrimination across multiple characteristics in England and the United States in middle- and older-aged adults.	Cohort (Longitudinal study)	N_1 (UK)_ = 8671 (ELSA), N_2 (US)_ = 7927 (HRS), Total = 16598		In England, perceived discrimination related to financial status and sexual orientation was more common, whereas in the US, more women perceived sex discrimination.	Country-specific and socioeconomic factors influence perceived discrimination prevalence, which are crucial for designing interventions to reduce such discrimination.
Meza et al.	Equitable suicide prevention for youth impacted by the juvenile legal system	USA	Provision of recommendations of priorities to promote health equity in suicide prevention for ethnoracially minoritized youth impacted by the juvenile legal system (JLS).	Review (perspective article)	Not applicable to the study.		Various touchpoints for suicide prevention care exist, and the Sequential Intercept Model, outlining community-based responses to individuals with mental and substance use disorders in the criminal justice system, can serve as a strategic planning tool. It helps outline equitable interventions across these touchpoints.	Addressing structural determinants of health is essential to prevent widening suicide disparities, moving beyond individual-level interventions.
Carlos et al.	Behavioral and psychosocial factors related to mental distress among medical students	USA	A comprehensive exploration on depression and suicidality in early-stage medical students, including an examination of suicidal history and the consideration of financial distress as a potential risk factor during medical school.	Cross-sectional	N = 134		Various individual-level and potentially modifiable risk factors were associated with higher scores on assessments of depressive symptoms and suicidal thoughts and behaviors.	The results underscore the critical need to implement tailored screening and offer resources for the well-being of the medical community.
Kleineberg-Massuthe et al.	Milieu-specific differences in symptom severity and treatment outcome in psychosomatic rehabilitation in Germany	Germany	Investigation of the association between social milieu and the severity of psychological symptoms, psychosocial impairments and symptom improvement over the course of rehabilitation.	Cross-sectional (Survey)	N = 2000		Patients from different backgrounds show notable variations in both BDI-II (depressive symptoms) and HEALTH-49 (assessment of impairments in specific areas).	Social milieu influences symptom severity, treatment access, and outcomes in psychosomatic rehabilitation. Considering milieu-specific sociocultural habits and therapeutic needs is crucial for planning and implementing therapy, enhancing equal access, quality, and effectiveness.
Mendes et al.	The psychological impact of Early Pregnancy Loss in Portugal: incidence and the effect on psychological morbidity	Portugal	Characterizing the psychological repercussions of early pregnancy loss (EPL) in relation to potential comorbidities, including complicated grief, depression, anxiety, and Post-Traumatic Stress Disorder (PTSD).	Cross-sectional (Survey)	N = 873 (women suffered a spontaneous loss)		Women who experienced loss within a month showed a higher proportion of comorbidity symptoms, with a significant gradual decrease over time in scores and proportion of clinical perinatal grief and PTSD.	Monitoring potential complex responses to the event of EPL is crucial to offer timely and suitable interventions for women requiring assistance.
Tseng et al.	Sex difference in the associations among risk factors with depression in a large Taiwanese population study	Taiwan	Investigation of the factors associated with depression and sex differences in a large Taiwanese population.	Cross-sectional	N = 121601		Women show a significant association with depression compared to men, and there are sex differences in the associated risk factors.	Early identification of factors linked to depression is crucial, but establishing causal relationships with risk factors remains inconclusive. Longitudinal studies are necessary to explore sex differences and incident depression.
Shang et al.	The relationship between alexithymia, depression, anxiety, and stress in elderly with multiple chronic conditions in China: a network analysis	China	Development of a network structure to investigate the connections between alexithymia, depression, anxiety, and stress in Chinese older adults with multiple chronic conditions.	Cross-sectional (survey)	N = 662		Difficulty Identifying Feelings (“DIF”) is a key element in the network of older adults with multiple chronic conditions (MCC), suggesting its importance for psychological interventions.	Healthcare professionals need to prioritize intervention for the psychological issues of older adults with alexithymia compared to those without alexithymia.
Garcia Nuñez et al.	Quality of life and associated factors in Swiss trans people: a cross-sectional study	Switzerland	Evaluation of trans people’s mental health, health-related QoL, psychological distress, self-esteem and the impact of life events occurred in the last six months on participants.	Cross-sectional (survey)	N = 30		The study found a negative correlation between life events’ impact and mental QoL, as well as between psychological distress and mental QoL. Additionally, a positive correlation was identified between self-esteem and mental QoL, with psychological distress and self-esteem serving as independent predictors of mental QoL.	Medical transition must not be viewed in isolation but must be embedded in the framework of integrative psychosocial support.
Mai et al.	The association between socioeconomic status and health-related quality of life among young and middle-aged maintenance hemodialysis patients: multiple mediation modeling	China	Study on the correlation between socioeconomic status (SES), illness perception, social functioning, and health-related quality of life (HRQoL) in young and middle-aged maintenance hemodialysis (MHD) patients.	Cross-sectional	N = 332 (young and middle-aged MHD)		Illness perception and social functioning mediate the SES-HRQoL association independently and cumulatively. SES correlates positively with HRQoL, while illness perception shows a positive correlation with social functioning.	Nurses should consider developing individual intervention program for young and middle-aged MHD patients with low SES, focusing on establishing targeted counseling and health education strategies corresponding to illness perception and social functioning to help patients improve their HRQoL.
Hu et al.	Decomposition and comparative analysis of depressive symptoms between older adults living alone and with others in China	China	Investigating and measuring the contributing factors that impact depression in older adults living alone vs. those living not alone.	Cross-sectional	N = 12197		Older adults living alone exhibit higher depressive symptom rates, primarily due to differences in socioeconomic status, education, income, sleep, and health status.	Addressing factors in the elderly living alone, could help developing precise intervention strategies to enhance the mental well-being of high-risk older adults.
Cogley et al.	Improving kidney care for people with severe mental health difficulties: a thematic analysis of twenty-two healthcare providers’ perspectives	Ireland	Investigating the barriers and facilitators to effective kidney care for people with severe mental health difficulties (SMHDs).	Qualitative (Semi-structured interviews with twenty-two healthcare professionals)	N_1_ = 14 (Physical) Professionals), N _2_ = 8 (mental professionals) Total N=22		Understanding individual challenges is crucial for supporting people with both SMHDs and kidney disease, enabling positive outcomes despite significant impairments and complex presentations.	Renal departments require multidisciplinary care, involving psychiatry, psychology, social work, and clinical nurse specialists. Coordination between renal and mental healthcare providers is vital for the safe and effective treatment of individuals with SMHDs.
Chen et al.	Disparities in the unmet mental health needs between LGBTQ+ and non-LGBTQ+ populations during COVID-19 in the United States from 21 July 2021 to 9 May 2022	USA	Evaluating the difference in UMHN between LGBTQ+ and non-LGBTQ+ during COVID-19.	Cross-sectional	N_1 = _81267 (LGBTQ+), N_2 = _722638 (non-LGBTQ+)		During COVID-19, LGBTQ+ faced increased UMHN risk. Disparities were noted across age groups, LGBTQ+ subtypes, and regions.	Tailored programs are crucial to address mental health vulnerabilities in diverse LGBTQ+ subgroups, with state-specific interventions. More research is needed on predictors and consequences of work alienation among nurses, requiring qualitative and quantitative studies to explore their understanding and experiences, enhancing knowledge for effective interventions.
You et al.	Status of work alienation among nurses in China: A systematic review	China	Systematically evaluating the status and distribution characteristics of work alienation among nurses.	Systematic Review and meta-analysis	Total of 12 studies with N (nurses) = 7265		Chinese nurses had moderate work alienation, particularly in specific categories.	Tailored programs are crucial to address mental health vulnerabilities in diverse LGBTQ+ subgroups, with state-specific interventions. More research is needed on predictors and consequences of work alienation among nurses, requiring qualitative and quantitative studies to explore their understanding and experiences, enhancing knowledge for effective interventions.
Mishu et al.	Exploring the contextual factors, behavior change techniques, barriers and facilitators of interventions to improve oral health in people with severe mental illness: A qualitative study	UK	To explore barriers and facilitators of oral health interventions to identify drivers of behavior change (capability, motivation, and opportunity).	Qualitative study (interview)	N = 12 (Intervention details were extracted from 12 intervention studies identified from a previous systematic review), Total 17 one-to-one interviews.		Individuals with SMI encounter obstacles in accessing dental care. The study explores barriers and facilitators for oral health interventions from the perspectives of people with SMI, caregivers, and service providers.	Comprehensive interventions are needed to improve oral health outcomes for individuals with SMI by addressing barriers and enhancing facilitators across various levels.

ELSA, English Longitudinal Study of Aging; HRS, US-based Health and Retirement Study; JLS, juvenile legal system; BDI-II, Beck Depression Inventory; HEALTH-49, Hamburg Modules for the Assessment of Psychosocial Health in Clinical Practice; MHD, maintenance hemodialysis; SES, socioeconomic status; HRQoL, health-related quality of life; SMHD, severe mental health difficulties; UMHNs, unmet mental health needs; SMI severe mental illness.

Various vulnerable populations are at an increased risk of mental health disorders such as depression and suicide. Particularly concerning suicide, it stands as the second leading cause of death for U.S. adolescents, with those in the juvenile legal system (JLS) being up to three times more susceptible. This crisis disproportionately affects Black and Latinx youth. A comprehensive approach involves diverting individuals to community treatment, preventing mental health deterioration during detention, supporting reentry post-detention, and assisting youth with mental health needs to prevent violations and new offenses. Addressing these intercepts is crucial for mitigating suicide risks in this vulnerable group (Meza et al.).

Contrary to the beliefs of the general population, physicians, also considered a vulnerable population, face an elevated risk of depression and suicide, a risk that becomes evident during medical school. Medical students exhibit higher rates of depression and suicidal thoughts than peers in other disciplines. Carlos et al. investigated mental distress and risk factors among US medical students, identifying poor sleep quality, impostor feelings, stress, and financial distress as key factors. Besides physicians, You et al. reported that Chinese nurses experience a moderate level of work alienation, marked by detachment from work due to infection risk, heavy workload, and persistent stress with consequences for both the individual and the organization. Recognizing this extent helps raise awareness among healthcare managers about the prevalent issue of work alienation among nurses and ensures the right for medical students to receive care for treatable mental health conditions.

Sex-specific differences, and disadvantaged sociocultural and economic status correlates with a higher prevalence of mental health issues including depression. Sex-specific differences are evident in depression, impacting clinical presentation, disease progression, and treatment response. Depression, affecting about 5% of adults globally, emerges as a common psychiatric issue. Associations between depression and risk factors vary by gender (Tseng et al.). When it comes to depression in the elderly, Hu et al . conducted a study in China to measure socioeconomic factors influencing depression in older adults living alone versus those living with others. The data revealed a higher prevalence of depressive symptoms in older adults living alone, primarily due to differences in socioeconomic status, marital status, years of school, self-reported local income, sleep, and health. Addressing these factors is crucial for developing targeted intervention strategies to improve the mental well-being of older adults at higher risk.

Moreover, disadvantaged sociocultural and economic status can impact mental illness treatment, particularly the psychosomatic rehabilitation sector, dedicated to preventing, treating, and compensating for chronic mental disorders. To detail inequalities in German psychosomatic rehabilitation, Kleineberg-Massuthe et al.‘s study reveals variations among patients from different social milieus concerning psychological symptom severity, psychosocial impairments, and treatment outcomes. Alongside structural care improvements, adapting and communicating services in ways more appealing to individuals from diverse social environments may be essential.

Targeted and customized mental health interventions are crucial for specific populations, aiming to enhance both their medical and psychiatric well-being. One such group facing notable health disparities is individuals with severe mental illness (SMI), leading to inferior outcomes and reduced life expectancy. Their oral health is notably worse, with a 3.4 times higher likelihood of complete tooth loss than the general population. While behavioral support interventions are generally effective in improving oral health behaviors, interventions for those with SMI encounter various challenges. Mishu et al.‘s study explores barriers and facilitators to oral health intervention engagement from the perspectives of people with SMI, caregivers, and service providers.

Turning attention to another demographic, it’s noteworthy that up to 25% of recognized pregnancies culminate in Early Pregnancy Loss, commonly referred to as miscarriage. This often precipitates enduring negative mental health responses, encompassing depression, anxiety, and Post-Traumatic Stress Disorder. Mendes et al.’s study pointed out that while clinical perinatal grief and PTSD scores significantly declined, a substantial number of women still experienced persistent clinical morbidities three years or more after the loss. Hence, timely monitoring and persistent intervention are crucial for those in need.

In a related context, approximately 4 million people with end-stage renal disease (ESRD) rely on renal replacement therapy, predominantly through maintenance hemodialysis (MHD). Studies highlight that socio-environmental, psycho-spiritual, and clinically relevant factors contribute to diminished health-related quality of life (HRQoL) in young and middle-aged MHD individuals. Nurses should develop tailored intervention programs, focusing on targeted counseling and health education strategies to address these factors and improve HRQoL (Mai et al.).

Another crucial aspect highlighted in the literature is the exploration of the connection between chronic conditions and mental health disorders. In this context, approximately 80% of individuals over 65 years old affected by multiple chronic conditions (MCC) experience impacted physical well-being, increased treatment costs, and are prone to psychological challenges such as depression, anxiety, and stress. Also, they exhibit a higher prevalence of alexithymia, affecting communication, disease management, recovery, and overall quality of life. Shang et al.’s study emphasizes Difficulty Identifying Feelings (DIF) as a significant psychological challenge, with the highest centrality and predictability across various alexithymia levels. Addressing the DIF tendency in older adults with MCC may improve other dimensions of alexithymia and alleviate symptoms of depression and anxiety in such communities.

In the realm of kidney care, individuals with severe mental health disorders (SMHDs) such as schizophrenia, bipolar disorder, and major depression face increased challenges. Cogley et al. brought to light additional barriers to kidney care for those with SMHDs, encompassing mental health challenges, motivation issues, cognitive difficulties, and mistrust of the healthcare system. Achieving effective kidney care requires an integrated “whole person” approach addressing the interplay between kidney disease and mental health.

Last not least there are papers investigating potential impacts of global trends and demographic shifts including COVID-19 pandemics, aging and the LGBTQ community, on mental health conditions. Chen et al.‘s US study during COVID-19 reveals a 2.27 times higher risk of unmet mental health needs (UMHNs) in LGBTQ+ individuals compared to non-LGBTQ+. Additionally, concerning associated socioeconomic factors, LGBTQ+ individuals are more likely to be younger (p < 0.001), unmarried, non-White, have lower education, lower household income, financial difficulties, and lack public and private insurance.

In parallel, another research study addressed the affected quality of life (QoL) due to life events during gender transition in transgender individuals. Garcia Nuñez et al.’s study on 30 transgender individuals highlighted significant mental health challenges, with psychological distress double that of cisgender norms. Trans individuals showed mental domain impairments in QoL, correlated negatively with life event impacts and psychological distress. Therefore, an integrative psychosocial support framework is essential, emphasizing that medical transition should not be isolated but considered holistically.

Finally, aging, as a global demographic challenge, can be associated with various cognitive and psychiatric disorders such as dementia, depression and suicides. Globally, the aging population is on the rise, with projections indicating that by 2050, individuals aged 65 and over will make up 24% and 21.4% of the UK and US populations, respectively. The health and well-being of older adults are significant policy priorities, and perceived discrimination is increasingly recognized as a risk factor for compromised healthy aging (Amirova et al.).

Studies presented here are of 14 published studies of Frontiers in the Research Topic of “*Disparities in Mental Health*”. It is to be hoped that many interesting scientific results will be reflected in mental health disparities worldwide.

## Author contributions

MM: Validation, Writing – original draft, Writing – review & editing, Investigation. HKo: Project administration, Supervision, Validation, Writing – review & editing. FM: Conceptualization, Data curation, Methodology, Supervision, Validation, Writing – review & editing. HKa: Writing – review & editing. LH: Supervision, Writing – review & editing.

